# Ellman's method is still an appropriate method for measurement of cholinesterases activities

**DOI:** 10.17179/excli2018-1536

**Published:** 2018-08-13

**Authors:** Seyed Vahid Shetab-Boushehri

**Affiliations:** 1 Department of Toxicology & Pharmacology, School of Pharmacy, International Campus, Iran University of Medical Sciences, Tehran, Iran; 2 Razi Drug Research Center, Iran University of Medical Sciences, Tehran, Iran

## ⁯

Dear Editor,

It has been recently argued that Ellman's method has not sufficient accuracy for measurement of cholinesterases (ChEs) activities (Komersová et al., 2007[3]; Sinko et al., 2007[6]; Pohanka et al., 2011[5]; Dingova et al., 2014[1]). This method was first introduced and described by Ellman and his colleagues and is based on thiocholine-derivative (acetyl- or butyryl) hydrolysis by ChEs (true or pseudo) and reaction of resulting thiocholine with thiol reagent 5,5'-dithiobis-2-nitrobenzoic acid (DTNB) and formation of 5-thio-2-nitrobenzoic acid (TNB) anion. Measurement of TNB ion absorption at 410 nm for pseudo-ChE and 440 nm for reticulocytic acetyl-ChE indirectly determines ChE activity (Ellman et al., 1961[2]). Some authors proposed that indoxylacetate is better substrate than acetylthiocholine (ATCH) as it does not react with oxime antidotes and thiol used for Ellman's method (Pohanka et al., 2011[5]). Moreover, DTNB which is unstable, interacts with free sulfhydryl groups in the sample, and may affect cholinesterase activity (Dingova et al., 2014[1]). Other researchers showed that oximes react with ATCH (oximolysis), producing thiocholine and consequently TNB ion (Sinko et al., 2007[6]). Some authors also mentioned that when the concentration of DTNB is far higher than the concentration of ATCH, hydrolysis rate of ATCH is decreased resulting in a lower measured ChE activity (Komersová et al., 2007[3]). 

Although above-mentioned problems have been stated about Ellman's method, each of them has its own logical solution. First, by simple appropriate dilution of sample, the thiol- and oxime-containing compounds in the sample are also diluted and hence the extent of reaction of DTNB with these substances is considerably reduced (Mohammadi et al., 2017[4]). Second, molar attenuation coefficients of indoxylacetate and TNB are 3900 M^-1^.cm^-1^ (Pohanka et al., 2011[5]) and 13600 M^-1^.cm^-1^ (Ellman et al., 1961[2]) respectively. Thus, the detection limit for measurement of ChE activity by DTNB is about 3.5 times lower than that by indoxylacetate. Third, for DTNB concentrations of 0.2-0.598 mM, the best ratio of DTNB/ATCH concentrations is 1.25-3.74 which results in optimum rate of ATCH hydrolysis by ChE (Komersová et al., 2007[3]).
